# Two Families with Normosmic Congenital Hypogonadotropic Hypogonadism and Biallelic Mutations in *KISS1R (KISS1 Receptor)*: Clinical Evaluation and Molecular Characterization of a Novel Mutation

**DOI:** 10.1371/journal.pone.0053896

**Published:** 2013-01-18

**Authors:** Frédéric Brioude, Jérôme Bouligand, Bruno Francou, Jérôme Fagart, Ronan Roussel, Say Viengchareun, Laurent Combettes, Sylvie Brailly-Tabard, Marc Lombès, Jacques Young, Anne Guiochon-Mantel

**Affiliations:** 1 Faculté de Médecine Paris-Sud UMR-S693, Univ Paris-Sud, Le Kremlin Bicêtre, France; 2 INSERM U693, IFR93, Le Kremlin Bicêtre, France; 3 Service de Génétique Moléculaire, Pharmacogénétique et Hormonologie, Hôpital Bicêtre, Assistance Publique-Hôpitaux de Paris, Le Kremlin Bicêtre, France; 4 Université Paris–Diderot, Paris 7, Paris, France; 5 Département d'Endocrinologie Diabétologie et Nutrition, Hôpital Bichat, Assistance Publique-Hôpitaux de Paris, Paris, France; 6 Faculté des Sciences, INSERM UMR-S757, Univ Paris-Sud, Orsay, France; 7 Service d'Endocrinologie et des Maladies de la Reproduction and Centre de Référence des Maladies Endocriniennes Rares de la Croissance, Hôpital Bicêtre, Assistance Publique-Hôpitaux de Paris, Paris, France; Innsbruck Medical University, Austria

## Abstract

**Context:**

*KISS1R* mutations have been reported in few patients with normosmic congenital hypogonadotropic hypogonadism (nCHH) (OMIM #146110).

**Objective:**

To describe in detail nCHH patients with biallelic *KISS1R* mutations belonging to 2 unrelated families, and to functionally characterize a novel *KISS1R* mutation.

**Results:**

An original mutant, p.Tyr313His, was found in the homozygous state in 3 affected kindred (2 females and 1 male) from a consanguineous Portuguese family. This mutation, located in the seventh transmembrane domain, affects a highly conserved amino acid, perturbs the conformation of the transmembrane segment, and impairs MAP kinase signaling and intracellular calcium release. In the second family, a French Caucasian male patient with nCHH was found to carry two recurrent mutations in the compound heterozygous state (p.Leu102Pro/Stop399Arg). In this man, pulsatile GnRH (Gonadotropin Releasing Hormone) administration restored pulsatile LH (Luteinizing Hormone) secretion and testicular hormone secretion. Later, long-term combined gonadotropin therapy induced spermatogenesis, enabling 3 successive pregnancies that resulted in 2 miscarriages and the birth of a healthy boy.

**Conclusion:**

We show that a novel loss-of-function mutation (p.Tyr313His) in the *KISS1R* gene can cause familial nCHH, revealing the crucial role of this amino acid in KISS1R function. The observed restoration of gonadotropin secretion by exogenous GnRH administration further supports, in humans, the hypothalamic origin of the gonadotropin deficiency in this genetic form of nCHH.

## Introduction

Congenital hypogonadotropic hypogonadism (CHH) results from abnormal gonadotropin secretion and is characterized by impaired pubertal development in both genders [1]–[4]. Identification of genetic abnormalities related to CHH has provided major insights into the pathways critical for the development, maturation and function of the reproductive axis [5], [6].

CHH may be caused by defective GnRH release or by a gonadotrope cell dysfunction in the pituitary. Loss-of-function mutations in the GnRH receptor were the first molecular defects to be found in patients with normosmic CHH (nCHH) [7]–[10] and are now recognized as one of the most frequent causes of familial autosomal recessive nCHH [11]. These patients are usually resistant to exogenous GnRH, but fertility can often be achieved through gonadotropin administration.

GnRH secretion is defective in most patients with nCHH and many nCHH-associated mutations leading to defective GnRH secretion have been described since ten years. These consist of loss-of-function mutations of *KISS1R*/*GPR54* [12]–[20] or its ligand Kiss [21], the *TAC3 (Tachykinin 3)* gene coding for neurokinin B and its receptor NK3R (Neurokinin 3 receptor), encoded by *TACR3 (Tachykinin receptor 3)* [22]–[26], and loss-of-function mutations of *GNRH1* [27], [28].

Only few patients with *KISS1R* mutations (NM_032551.4) have so far been reported [12]–[20], and the description of new cases is thus needed to define the phenotypic range of this genetic form, as well as the mechanism underlying the gonadotropin deficiency. Characterization of new mutations can also help to unravel the functional anatomy of this receptor.

Here we describe a new family with an original deleterious mutation carried in the homozygous state. We have undertaken to evaluate the functional consequences of this mutation. To this aim, we studied two well known KISS1R activated cascades. The phospholipase C pathway was assessed by measuring intracellular calcium mobilization while the MAP kinase pathway was tested by analyzing both the phosphorylation status of ERK1/2 by means of western blot analysis and the transactivation of serum responsive elements driven reporter luciferase gene. We also provide a detailed description of a male nCHH patient with 2 recurrent mutations carried in the compound heterozygous state but in a previously undescribed genetic combination, and we reinforce the concept that CHH in this genetic form is due to GnRH deficiency.

### Case reports

#### Family 1

**Figure 1:**
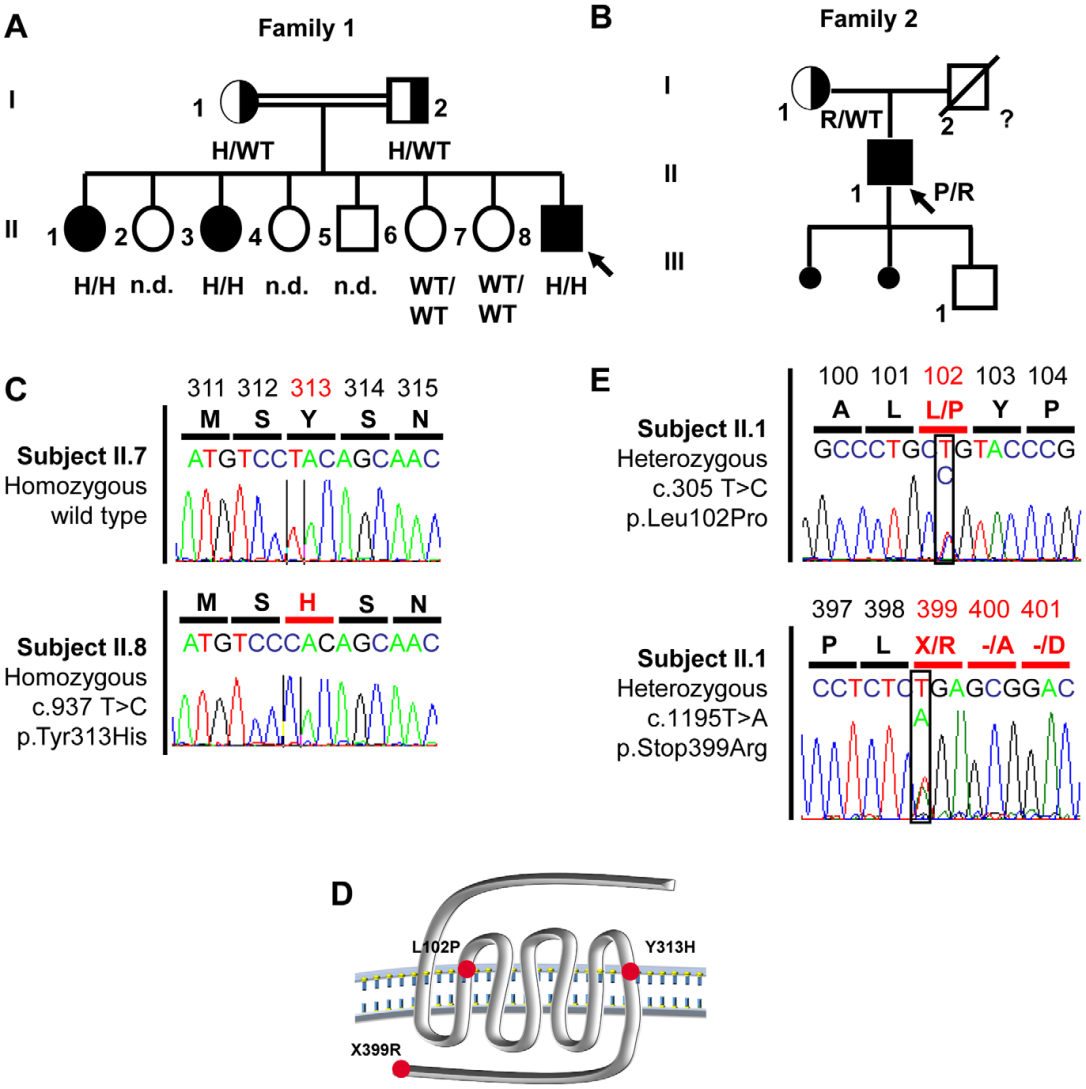
Family pedigrees and corresponding *KISS1R* mutations. **Panel A.** Pedigree of the family with homozygous *KISS1R* c.937T>C mutation. The proband, subject II-8 (arrow), and his two affected sisters, subjects II-1 and II-3, were homozygous for the c.937T>C mutation. The unaffected mother (I-1) and father (I-2) were heterozygous for the same mutation. Two unaffected sisters, II-6 and II-7, carried two wild-type alleles. This missense mutation (p.Tyr313His, (H)) is located in the seventh transmembrane segment. Squares represent males and circles females. Solid symbols indicate affected subjects and half-shaded symbols unaffected heterozygous. **Panel B.** Pedigree of the family with compound heterozygous *KISS1R* mutations c.305T>C and c.1195T>A. The proband, subject II-1 (arrow), was a compound heterozygote for the *KISS1R* mutations c.305T>C and c.1195T>A. The unaffected mother (I-1) was heterozygous for the c.305T>C mutation. The unaffected father was deceased and therefore unavailable for genetic analysis. The c.305T>C substitution produces a missense mutation (p.Leu102Pro, (P)) in the second transmembrane segment of KISS1R. The c.1195T>A substitution abolishes the natural stop codon (p.Stop399Arg, (R)). Small black circles indicate miscarriages. **Panel C.** Results of automatic DNA *KISS1R* sequencing encompassing the c.937T>C homozygous mutation in the propositus, compared to an unaffected control (lower panel). Inframe amino acids are indicated above each sequence. **Panel D.** Schematic representation of *KISS1R* and location of the mutations. **Panel E.** Results of automatic DNA *KISS1R* sequencing encompassing the c.305T>C and c.1195T>A compound heterozygous mutations in the propositus. Inframe amino acids are indicated above each sequence.

The family 1 propositus (subject II-8, Fig. 1A) was a 55-year-old man originating from Portugal who was referred in 2004 to the Endocrinology and Reproductive Diseases Department of Bicêtre Hospital for gynecomastia and hypogonadism. He stated that he had never experienced pubertal development or had sexual intercourse, and that his unaffected parents were first cousins. He had typical hypogonadism, with small intrascrotal testes (2 and 3 mL right and left, respectively), near-normal pubic hair (P4), and a penis length of 4 cm. His height was 173 cm and his weight 76 kg. The karyotype was normal (46, XY). His sense of smell was normal on olfactometry [29]. Pituitary and olfactory bulb magnetic resonance imaging (MRI) and renal sonography were also normal.

At initial evaluation his serum testosterone, FSH (Follicle stimulating Hormone), LH and inhibin B levels were very low, (Table 1). After a single bolus of 100 µg of GnRH intravenously (IV), LH levels rose from a baseline of 0.2 to a peak of 5.1 IU/L, and FSH rose from 0.1 to 4.1 IU/L.

**Table 1:** Clinical and hormonal characteristics in affected patients with *KISS1R* mutations of two kindred.

Family	1						2
	nCHH					normal	nCHH
**Subjects (see ** **Fig. 1** **)**	**II-1**	**II-3**		**II-8**		**II-7**	**II-1**
**Genotype**	c.[937T>C]+c. [937T>C]					[ = ]+[ = ]	c.[305T>C]+[1195T>A]
**Aminoacid change**	p.[Tyr313His]+[Tyr313His]					WT	p. [Stop399Arg]+[Leu102Pro]
**Sex/age at diagnosis**	F/30	F/32		M/55		F	M/19
**Reproductive phenotype at diagnosis**	Primary amenorrhea, absent breast development	Primary amenorrhea, absent breast development		Penis: 4 cm Mean TV: 2.5 mL		Hormonal evaluation at menopausal age	Penis: 3.5 cm Mean TV: 2 mL
**Reproductive phenotype in heterozygous parents**	normal						normal
**T (ng/mL)**	-	-		0.2		-	0.25
**E2 (pg/mL)**	15	13		-		10	
**FSH (IU/L) Basal/stimulated ^(1)^**	1.3	1.2		0.1/4.1		45	0.2/1.1
**LH (IU/L) Basal/stimulated^(1)^**	1.5	0.8		0.2/5.1		27	0.15/1.3
**Inhibin B (pg/mL)**	-	-		17		-	25
**Other anterior pituitary dysfunctions^(2)^**	no	no		no		-	no
**Anosmia or hyposmia^(3)^**	no	no		no		**-**	no

(1) GnRH, 100 μg IV; (2) Basal Prolactin, FT4 and TSH and GH and cortisol under insulin hypoglycemia challenge test; (3) olfactometry Normal range in adults, LH : 2.8-7.1 IU/L; FSH : 2.4-7.0 IU/L, T : 2.8-9.0 ng/mL, E2 : 24-90 pg/mL (early follicular phase).

His two affected sisters (subject II-1 and subject II-3, Fig. 1A), who were initially evaluated at the ages of 30 and 32 years, respectively, also had complete hypogonadism and said they had a normal sense of smell. At diagnosis, both sisters had absent breast development but near-normal pubic hair (P4). Menarche had never occurred. Initial hormone assays revealed very low serum estradiol, LH and FSH levels in the two sisters (Table 1), both of whom had a normal karyotype (46, XX). Recent hormonal evaluation of an unaffected sister at 52 years of age showed normal levels for age.

#### Family 2

**Figure 2:**
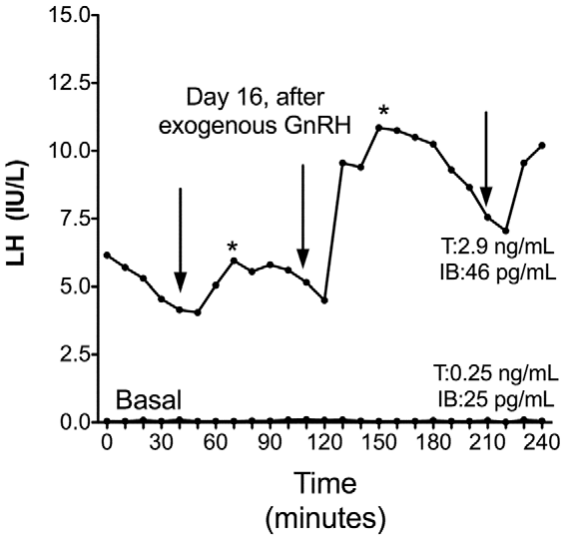
Pattern of LH secretion in a man with complete normosmic CHH due to *KISS1R* mutations (subject II-1, family 2). In this individual with very low and nonpulsatile basal LH levels, LH pulsatility was restored by pulsatile GnRH administration. LH secretion was evaluated for 4 hours before GnRH treatment and also on day 16 of pulsatile GnRH administration. *Arrows* indicate the GnRH boluses and *asterisks* denote detectable pulses, as analyzed with Thomas' algorithm [7], [27]. Plasma testosterone (T) levels, which were very low before GnRH treatment, increased after pulsatile GnRH administration in this man, indicating testicular stimulation. Conversion to SI units: testosterone, nanograms per milliliter (x 3.467 = nanomoles per liter). The testosterone normal range in men is 2.8–9.0 ng/ml. IB: low serum inhibin B also increased after pulsatile GnRH administration.

The family 2 proband (subject II-1, Fig. 1B) was a French Caucasian man born to non consanguineous eugonadal French parents. He was initially referred to our department at age 19 years for absent pubertal development. Physical examination showed typical signs of hypogonadism, with small intrascrotal testes (2 mL) and a penis length of 3.5 cm. His height was 185 cm and his weight 73 kg. His karyotype was normal (46, XY). He had very low levels of serum testosterone (0.25 ng/mL), serum LH (0.15 IU/L) and FSH (0.2 IU/L), which did not respond to GnRH challenge (Table 1). He was again seen at age 27 years, 3 months after he had decided to stop testosterone enanthate therapy. His mean testicular volume was still low (3 mL), as were his testosterone (T), gonadotropins (T: 0.25 ng/mL, LH: 0.12 IU/L, FSH: 0.17 IU/L), and inhibin B levels (25 pg/mL). At that time, an analysis of basal LH secretion overnight at 10-min intervals for 4 h showed very low levels of this gonadotropin and a nonpulsatile pattern (Fig. 2). On day 16 of pulsatile GnRH administration (100 ng/kg/pulse, every 60 min, sc), pulses of LH release were detected, occurring synchronously with the GnRH boluses (Fig. 2) and concomitantly with an increase in serum testosterone and inhibin B levels.

Androgen therapy (testosterone enanthate, 250 mg every 3 weeks, intramuscular injections) was then resumed, leading to satisfactory libido. One year later his testicular volume was still low (3 mL) and he was azoospermic. As he wished to have children, he was treated for practical reasons with combined gonadotropin therapy (recombinant human FSH, 150 IU three times a week, sc) and hCG (1500 IU three times a week, sc) in order to induce spermatogenesis. This combined therapy led to a gradual but marked increase in testicular volume (10 and 12 mL left and right, respectively) and normalization of testosterone and inhibin B levels. After 22 months of treatment the sperm count was 19×10^6^/mL.

His partner became pregnant twice within one year of starting this treatment, but both pregnancies ended in early miscarriage. A healthy boy was born after a third pregnancy.

Pituitary and olfactory bulb magnetic resonance imaging (MRI) were normal in this patient, and olfactometry [29] showed a normal sense of smell.

All the affected patients in family 1 and family 2 had normal renal echography, no craniofacial abnormalities, normal circulating iron, ferritin, IGF1 and prolactin levels, and normal pituitary, adrenal and thyroid function. No other phenotypic abnormalities were found.

## Methods

All the participants gave their written informed consent for hormonal exploration and genetic analyses, in keeping with the French Bioethics Law and the Declaration of Helsinki, and after approval by the Bicêtre Hospital ethics committee (Comité de protection des personnes Ile de France, Hôpital Bicêtre).

### Hormone assays

We measured serum levels of LH, FSH, inhibin B, testosterone and estradiol by immunoradiometric assay, enzyme-linked immunosorbent assay, or radioimmunoassay, respectively, as previously reported [27], [24], [30]. The detection limits of the LH and FSH assays were respectively 0.1 IU/L and 0.1 IU/L. The intra- and interassay coefficients of variation were respectively 1.5% and 5.2% for LH, and 2.7% and 5.5% for FSH. Endogenous LH secretion, analyzed with Thomas' algorithm, was measured overnight at 10-minute intervals during 4 hours, as reported elsewhere [7], [27].

### DNA analysis

Genomic DNA was extracted from white blood cells by using standard procedures.

*KISS1R* coding exons 1, 2, 3, 4 and 5 and intron-exon junctions were amplified by PCR (Polymerase chain reaction) and sequenced as previously described, with minor modifications [12]. Sequence variations were found on both strands and confirmed in a separate PCR analysis.

*GNRHR (Gonadotropin Releasing Hormone Receptor)*, *GNRH1*, *TAC3*, *TACR3*, *KISS1*, *FGFR1 (Fibroblast Growth Factor Receptor 1)* and *PROK2/PROKR2 (Prokineticin 2/Prokineticin receptor 2)* were also analyzed as previously described, with minor modifications [7], [31], [32], [27], [33], [24], [21].

### Modeling studies

A three-dimensional model of KISS1R was generated by homology with the Modeller package (version 9.10) [34], using as template the crystal structure of the kappa opioid receptor (OPRK1), a hepta-transmembrane protein [35]. *KISS1R* mutants were generated by using the O package [36]. Statistics, calculated with Molprobity [37], showed that 99.0% of the residues in the Ramachandran analysis plot were in the most favored or allowed regions, and that side-chain stereo parameters were within the range of or better than the statistics derived from a set of crystal structures of at least 2.0 Å resolution (see supplemental data Fig. S1). In addition, the PROSAII program gave a combined Z-score (Cβ and surface potentials) of −3.34, a value within the range of structured proteins. These results suggested that the protein was of good quality and suitable for analysis.

### Functional studies

#### Directed mutagenesis

The KISS1R p.Tyr313His mutant was reproduced by site-directed mutagenesis using the pcDNA3.1+ plasmid encoding human KISS1R (*Missouri S&T cDNA Resource Center*) and the QuickChange Stratagen II kit (*Stratagen*, La Jolla, CA). Clones were verified by sequencing.

#### Intracellular calcium analysis

A fluorometric calcium mobilization assay was used to assess the response of the KISS1R mutant to the kisspeptin-10 (Kp-10) decapeptide (Sigma-Aldrich, Saint-Quentin Fallavier, France), a potent KISS1R agonist [15]. This assay was capable of detecting KISS1R activity: calcium flux above baseline for wild-type KISS1R was detected at nanomolar ligand concentrations, with maximal flux achieved at 10^−7^ M.

For intracellular calcium analysis, 15×10^4^ COS-7 cells were plated on standard 25-millimeter-diameter coverslips in standard 6-well plates 24 hours before transfection with 500 ng of empty (PCDNA3.1 with no inserted sequence), wild-type or mutant KISS1R-plasmid per well, in the presence of 1.25 µl of Lipofectamine™ 2000 (Lifetechnologies, Saint Aubin, France) in a final volume of 2 ml of Opti-MEM medium (Lifetechnologies). Six hours after transfection the medium was removed and the cells were grown in standard medium. Forty-eight hours after transfection the cells were washed once in PBS and then incubated in modified Hank's buffer saline solution (HBSS) containing Fura-2 AM at a final concentration of 3 µM, for 20 minutes at room temperature. The buffer was then removed and the cells were incubated in HBSS without Fura-2 for 5 minutes. Calcium imaging was then performed with an inverted fluorescence microscope, as previously described [38]. Fluorescence images were collected with a CCD camera (Princeton, USA), digitized and integrated in real time by an image processor (Metafluor, Princeton, USA). A field with 20 to 30 cells was chosen for treatment with kisspeptin 10 (Kp-10) 10^−9^ or 10^−7^ M. Excitation wavelengths were 340 nm (free Fura-2) and 380 nm (bound Fura-2). Emission was read at 510 nm. The 340/380 nm ratio was recorded for 10 minutes. For each cell within the field, we determined the area under the curve (AUC) after stimulation with Kp-10, using GraphPad Prism 5 software.

#### Western blot analysis

For western blot analysis (3 independent experiments), 15×10^4^ HEK-293 cells were plated in standard 6-well plates 72 hours before transfection with 1 µg of wild-type or mutated vector per well, in the presence of 2.5 µl of Lipofectamine™ 2000 (*Invitrogen*) in a final volume of 2 ml of Opti-MEM medium (*Invitrogen*). Six hours after transfection the medium was removed and the cells were grown in standard medium. Forty-eight hours after transfection the cells were treated with Kp-10 (*Sigma-Aldrich*) 10^−8^ M or 10^−6^ M for 5, 10, 20, 30 and 60 minutes. Cells were harvested and lysed, and 30 µg of total protein was deposited on a 12% polyacrylamide gel, separated by electrophoresis, and transferred to a PVDF membrane. The membrane was incubated in blocking solution containing a primary antibody (anti-phospho-p44/p42 MAP kinase; Cell Signaling, USA) at a final concentration of 1/1000 for one hour at room temperature, then in blocking solution containing a secondary antibody (anti-rabbit coupled to peroxidase, 1/15 000; Vector Labs) for one hour at room temperature. The signal was revealed by using ECL-Plus (*GE Healthcare Vélizy, France*) on autoradiographic film. Normalization was based on the use of an anti-p44/42 MAP kinase antibody (*Cell Signaling*, Ozyme, Saint-Quentin en Yvelines, France) at a final concentration of 1/1000, following the same steps. The signal was quantified with Quantity One software (*Biorad Marnes La Coquette, France*). Each value of this ratio was expressed as a percentage of the T0 value (untreated cells). Molecular weight was determined with Precision plus Protein Standards Dual color (Promega). Apparent molecular weight of ERK1/2 (p-42/p-44 kDa) was between the 37 and 50 kDa standards as expected.

#### Luciferase reporter gene assay

Kisspeptin protein-coupled receptors signal through several second-messenger pathways, including the phosphoinositide and MAP kinase pathways (ERK 1/2) [39]–[41]. We thus used the luc2P/SRE/Hygro plasmid (*Promega*, Charbonnières Les Bains, France), which can induce luciferase production in response to MAP kinase activation, as a reporter gene system [42]. For SRE activation analysis, 10^5^ HEK-293 cells were plated in standard 24-well plates 48 hours before transfection with 50 ng of empty vector PCDNA3.1, wild-type or mutated KISS1R-plasmid, 100 ng of SRE-Luciferase-plasmid and 100 ng of ß-galactosidase-plasmid (for normalization) in the presence of 0.5 µl of Lipofectamine™ per well, in a final volume of 500 µl of Opti-MEM medium. Six hours after transfection the medium was removed and the cells were grown in standard medium. The following day, cells were then incubated with increasing concentrations (from 10^−10^ to 10^−6^ M) of Kp-10 for 6 h before harvesting for ß-galactosidase and luciferase assay, as previously described [26]. Luciferase activity was measured with a luminometer (*Victor*, *Perkin Elmer*, Courtaboeuf, France). Curves were fitted with data from at least three independent experiments performed in triplicate.

#### Statistical Analysis

Intracellular calcium values are expressed as means +/− SEM. The AUCs for each condition (WT or p.Tyr313His KISS1R transfected cells) were compared by using the Kruskall Wallis test with Dunn's post test to compare each pair of conditions. GraphPad Prism 5 software was used for all statistical analyses. Differences were considered significant at p<0.05.

## Results

### Molecular analyses

The *GNRH1*, *GNRHR*, *KISS1*, *TAC3*, *TACR3*, *FGFR1*, *PROK2* and *PROKR2* exons and intron–exon boundaries were identical to the reference sequences in all the affected members of both 2 families.

**Figure 3:**
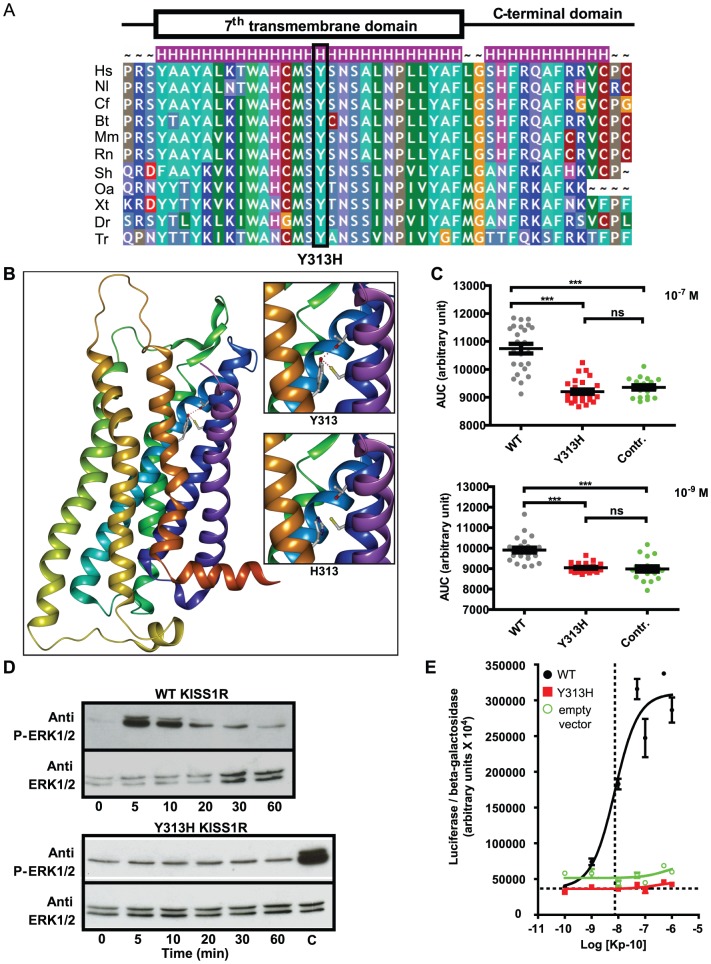
Modeling and functional consequences of the p.Tyr313His *KISS1R* mutation. Panel A. Evolutionary conservation of Tyr313. Tyr313 is totally conserved among KISS1R homologs. Hs: Homo sapiens; Nl: Nomascus leucogenys; Cf: Canis familiaris; Bt: Bos taurus; Mm: Mus musculus; Rn: Rattus norvegicus; Sh: Sarcophilus harrisii; Oa: Ornithorhynchus anatinus; Xt: Xenopus tropicalis; Dr: Danio rerio; Tr: Takifugu rubripes. The substitution is indicated below. **Panel B.** Modeling of the transmembrane region of KISS1R. Tyrosine 313 (and its substitution by a histidine) are located at the bottom of the putative binding pocket for kisspeptin. Tyr313 is in a suitable position to form two hydrogen bonds with Cys95 and Thr99 (see upper insert), both located in the second transmembrane segment. The His substitution abolishes these interactions (lower insert). **Panel C.** Variation of intracytoplasmic calcium concentrations in WT, Tyr313His (Y313H) mutated KISS1R and empty vector. Transfected cells were stimulated by kisspeptin (10^−7^ M and 10^−9^ M): we observe a very significant decrease in calcium release in mutant-KISS1R transfected cells (***p<0.0001). Each point represents the area under the curve (AUC) for an individual cell. WT: black circles, Y313H: red squares, empty vector green circles. **Panel D.** ERK1/2 phosphorylation in WT and Y313H HEK-293 transfected cells. The upper panel shows the marked increase in ERK1/2 phosphorylation in cells transfected with the wild-type KISS1R, after 5 minutes of Kp-10 stimulation. The lower panel shows the absence in ERK1/2 phosphorylation when cells were transfected with the vector containing Y313H-mutated KISS1R. C: positive control: WT KISS1R stimulated by kisspeptin after 5 minutes. Representative figure of 3 independent experiments. **Panel E.** Kp-10 dose-response of the luc2P/SRE reporter. Increasing concentrations of Kp-10 led to a gradual increase in the luciferase activity of wild-type KISS1R (black circles). In contrast, the mutant KISS1R (red squares), like the empty vector (green circles), did not enhance luciferase activity.

The family 1 propositus and 2 affected sisters were found to carry a **homozygous** ***KISS1R*** **mutation (c. 937T>C)** (subjects II-1, II-3 and II-8, Fig. 1A and Fig. 1C). The mutation was found in the heterozygous state in the patient's unaffected mother (subject I-1, Fig. 1A) and father (subject I-2, Fig. 1A). This substitution results in a missense mutation (p.Tyr313His) affecting a highly conserved amino acid (Fig. 3A) located in the seventh transmembrane segment (Fig. 1D). This mutation was not found by genomic sequencing in 200 chromosomes from Caucasian eugonadal subjects.

In the family 2 propositus II.1 (Fig. 1B) we found a **compound heterozygous** ***KISS1R*** **mutation (c.305T>C and c.1195T>A)**. C.305T>C is a recurrent missense mutation (p.Leu102Pro) in the second transmembrane segment of KISS1R (Fig. 1D and Fig. 1E). It has previously been reported in the homozygous state in Arab-Muslim CHH patients, and was characterized as a loss-of-function mutation by Tenembaum et al. [17]. The same mutation was found in the heterozygous state in the unaffected mother.

The c.1195T>A substitution, abolishes the stop codon (p.Stop399Arg) (Fig. 1D). This mutation has previously been reported in the compound heterozygous state in an AfroAmerican man with nCHH, in association with the nonsense p.Arg331stop mutation described by Seminara et al. [13] and characterized as deleterious.

Neither of these mutations was found by genomic sequencing in 200 chromosomes from Caucasian eugonadal subjects.

### Modeling and functional studies of the missense mutations

Using the Pipealign program, we found that the kappa opioid receptor OPRK1, for which the crystal structure is available (protein database identity number 4DJH), displayed the strongest homology with KISS1R in the hepta-transmembrane protein family (34% sequence identity). Moreover, OPRK1 is a G-coupled receptor whose natural ligand is dynorphin A or B, peptides with lengths of 13 and 17 amino acids, similar to those of kisspeptin (10, 13 or 14 amino acids). This receptor is thus a good candidate structural template for constructing a KISS1R homology model. This model revealed that Tyr313, which is highly conserved among species, is located in the seventh transmembrane segment (Fig. 1D, Fig. 3A and Fig. 3B) and is in a suitable position to form two hydrogen bonds with Cys95 and Thr99, both of which are located in the second transmembrane segment (Fig. 3B and supplemental data Fig. S2). These peculiar interactions suggest that Tyr313 might stabilize the cohesion of the transmembrane bundle. Moreover, Tyr313 is located at the bottom of the putative binding pocket for kisspeptin and could therefore participate in its anchoring. The shorter side-chain of histidine in the p.Tyr313His mutant receptor, being no longer able to form a hydrogen bond with Cys95 and Thr99, might undermine the cohesion of the transmembrane bundle.

#### Intracellular calcium flux variations

Fig. 3C shows individual fluorescence variations (AUC values) after Kp-10 administration to cells transfected by empty vector, WT KISS1R or the Tyr313His mutant. Each point of the graph represents one cell in the explored field. The mean AUC was significantly lower in Tyr313His-KISS1R transfected cells than in WT cells after stimulation with Kp-10 at both 10^−7^ M and 10^−9^ M (p<0.0001). No difference in AUC was observed between control (empty vector) and Tyr313His-KISS1R.

#### MAPK pathway activation

WT-KISS1R transfected cells showed an increase in ERK1/2 phosphorylation 5 minutes after treatment with 10^−8^ M Kp-10 (Fig. 3D higher panel). No activation of ERK1/2 phosphorylation was observed in Y313H-KISS1R transfected cells, at either 10^−8^ M (Fig. 3D, lower panel) or 10^−6^ M Kp-10 (data not shown).

To quantify and compare the stimulatory effect of Kp-10 on wild-type and mutant KISS1R, we constructed dose-response curves for HEK-293 cells in the SRE luciferase assay (Fig. 3E). The wild-type KISS1R plasmid led to strong stimulation of the SRE-luciferase plasmid, with an average EC50 of 9.3×10^−9^ M. In contrast, the Tyr313His-KISS1R plasmid failed to stimulate the reporter plasmid (Fig. 3E).

Thus, our functional analyses clearly showed that the Tyr313His-KISS1R mutant failed to stimulate the p44/42 MAP kinase pathway, contrary to the wild-type receptor, which caused a clear dose-response in the presence of Kp-10.

## Discussion

The first aim of this study was to describe the clinical and hormonal features of a new consanguineous family with nCHH and a biallelic *KISS1R* mutation, and to functionally characterize this new mutation. In this family, only subjects with mutations in both alleles had a nCHH phenotype, in keeping with the mainly autosomal recessive transmission in families with this genetic form of nCHH [12]–[20]. Interestingly, the proband of this family still had a complete gonadotropin deficiency at age 55, indicating that CHH in this family is irreversible, as appears to be case of many patients with this genetic form [43].

Investigation of the second family also showed autosomal recessive transmission and demonstrated that the gonadotropin deficiency could be corrected by pulsatile GnRH administration, lending further weight to the concept originally proposed by Seminara et al [13], [16], namely that the gonadotropin deficiency in these patients is secondary to their GnRH deficiency as been demonstrated in several animal models [44]. These data therefore further support the idea that loss of KISS1R function in pituitary gonadotropic cells does not have clinically significant consequences in humans in agreement with the absence of KISS1R expression in gonadotrope cells reported in monkeys [45]. Similarly, restoration of spermatogenesis and fertility after gonadotropin therapy in this patient shows that KISS1R loss of function has no direct effect on testicular function.

Another feature of note is that the partner of the family 2 proband had two miscarriages before giving birth to a healthy boy. Indeed, miscarriages have also been reported by Pallais et al. in this setting [16], suggesting that the KISS1R mutation may affect placentation.

The integrity of other ante-pituitary functions in the patients investigated here also shows that loss of KISS1R only affect gonadotrope axis. Of particular interest, corticotrope axis was not affected despite immunoexpression of KISS1R in pituitary cortocotropes cells as reported in non human primates [45].

Investigations of the gonadotropic axis, performed relatively late in the disease course, suggested that nCHH is not reversible in these patients.

The propositus and affected sisters of family 1 carried a new missense mutation, p.Tyr313His, in the homozygous state. Molecular modeling suggested that this mutation, affecting a conserved amino acid in the seventh transmembrane domain, alters the conformation of this domain. The deleterious nature of this variant was confirmed by functional analyses showing impaired intracellular calcium release and defective MAP kinase-mediated KISS1R signaling.

We used the kappa opioid receptor as the reference structure for our modeling study [35], because its sequence is closer to that of KISS1R compared to rhodopsin, the usual reference structure for seven-transmembrane receptors [19].

The lack of stabilizing contacts between the second and seventh transmembrane segments in the Tyr313His mutant receptor, through hydrogen bonds between Cys95, Thr99 and His313, is likely responsible for the loss of capacity to transduce the ligand binding signal and/or the ability to adopt the receptor conformation required for high kisspeptin affinity and binding. Alternatively, the Tyr313His mutation could lead to KISS1R misfolding or dysfunction as previously reported for the p.Phe272Ser KISS1R mutation [19].

In the second family, of Caucasian origin, we found the recurrent mutation p.Leu102Pro, previously described in the homozygous state [17], but present here in the compound heterozygous state. It was associated, in a previously undescribed configuration, with another recurrent mutation, p.Stop399Arg. The latter was first described by Seminara, also in the compound heterozygous state with mutation p.Arg331Stop [13]. Its deleterious nature has been demonstrated in terms of IP3 release [13].

In conclusion, we describe two new families with nCHH and *KISSIR* mutations. One mutation is original and reveals the crucial role of tyrosine 313 located in transmembrane domain 7. In the other family, recurrent mutations described in other ethnic groups were associated in an original format. The observed pituitary and gonadal response to pulsatile GnRH administration supports, in humans, the hypothesis that disruption of the gonadotropic axis due to KISS1R loss of function occurs exclusively in the hypothalamus.

## Supporting Information

Figure S1 **Rammachandran plots for human KISS1R tridimensional model.** Statistics, calculated with Procheck, showed that 99.0% of the residues in the Ramachandran plot were in the most favored or allowed regions, and that side-chain stereo parameters were within the range of or better than the statistics derived from a set of crystal structures of at least 2.0 Å resolution.
(PPT)

Figure S2 **Human KISS1R tridimensional model.** Panel A: Tyr313, located in the middle of 7th transmembrane segment, is pointing at the hydrophobic core. The hydroxyl group of the tyrosine residue may form 2 hydrogen bonds with Cys95 and Thr99 both located in the 2nd transmembrane segment and stabilize cohesion of transmembrane bundle. These residues may participate in the docking of kisspeptin. Panel B: Focus on the mutated amino-acid residue: on the left, hydroxyl group of Tyr313 residue probably make hydrogenic bond with Cys95 (2.75 Å) and Thr99 (2.62 Å) *(Normal Hydrogenic bond range is 2.7 to 3.2 Å)*. On the right: Imidazole group of histidine mutant residue in position 313 is not able to form hydrogen bond with Cys 95 and Threonine 99 both located in the 2nd transmembrane segment and therefore impair cohesion of transmembrane bundle.
(PPT)
