# Supplementary material for: Two Families with Normosmic Congenital Hypogonadotropic Hypogonadism and Biallelic Mutations in KISS1R (KISS1 Receptor): Clinical Evaluation and Molecular Characterization of a Novel Mutation
Source: PLoS One. 2013 Jan 18;8(1):e53896. doi: 10.1371/journal.pone.0053896 (PMC3548821; doi:10.1371/journal.pone.0053896)
Supplement: Figure S1 — Rammachandran plots for human KISS1R tridimensional model. Statistics, calculated with Procheck, showed that 99.0% of the residues in the Ramachandran plot were in the most favored or allowed regions, and that side-chain stereo parameters were within the range of or better than the statistics derived from a set of crystal structures of at least 2.0 Å resolution. (PPT) [file pone.0053896.s001.ppt]

## Slide 1
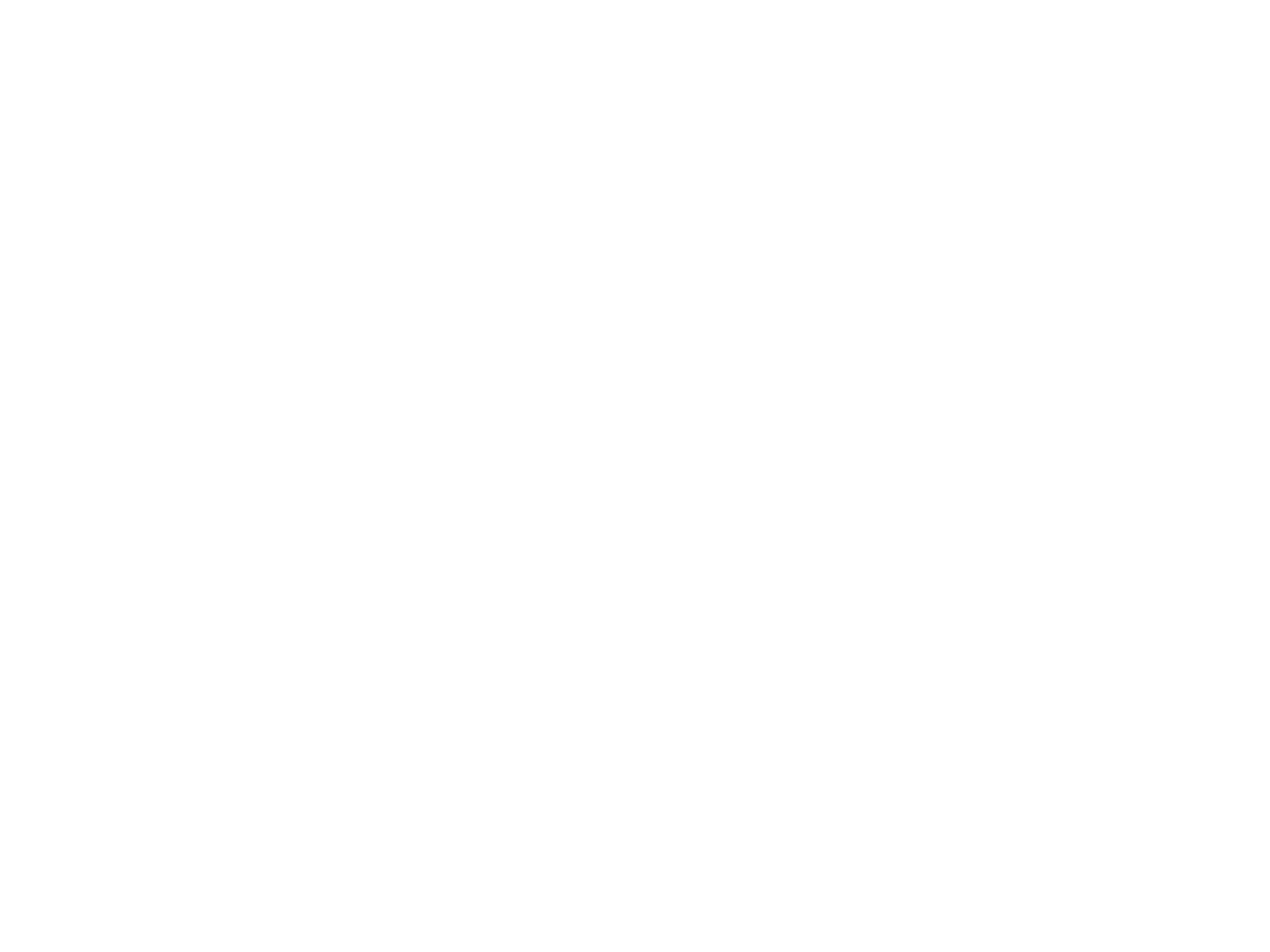

#

## Slide 2
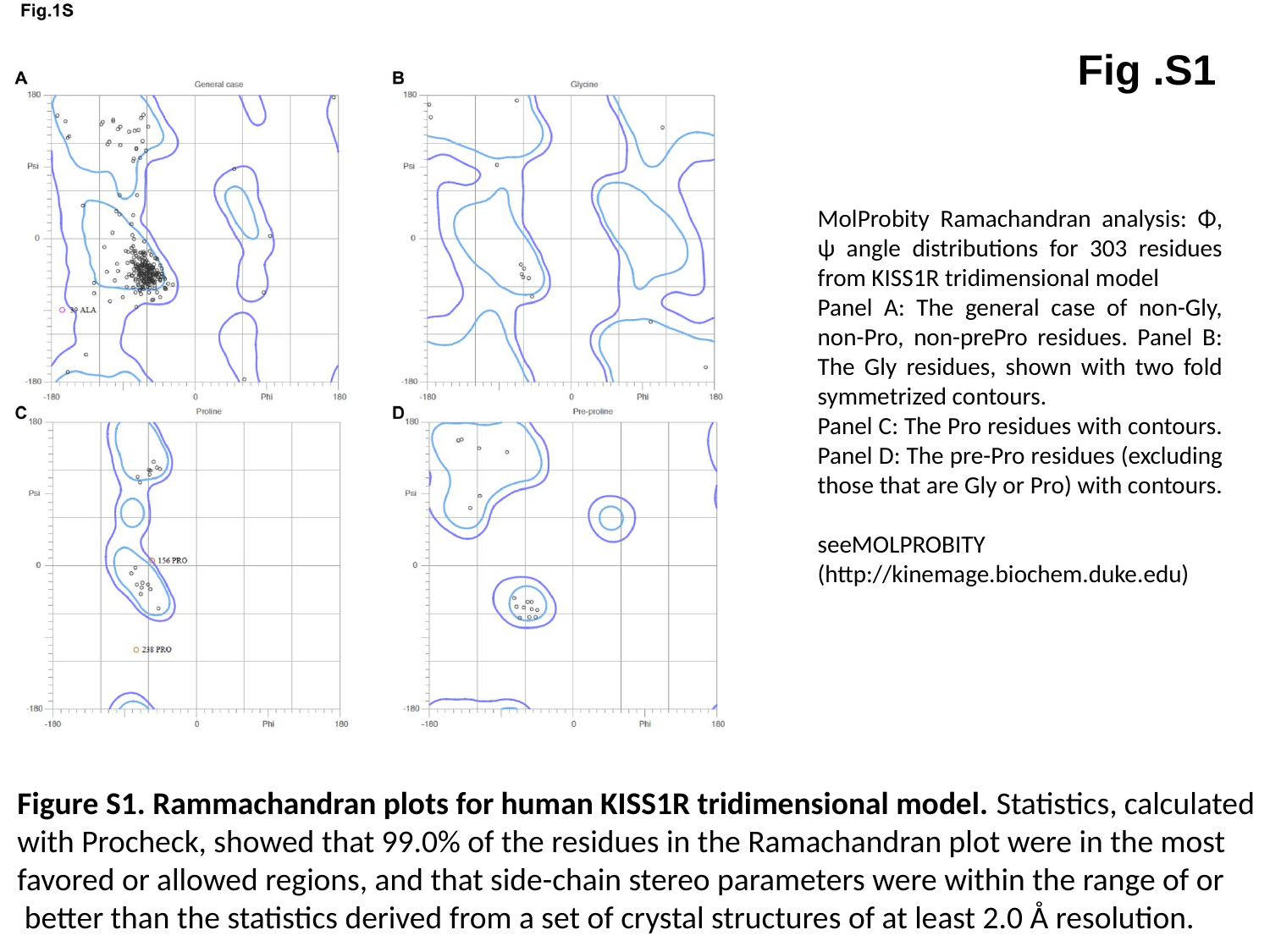

Fig .S1
MolProbity Ramachandran analysis: Φ, ψ angle distributions for 303 residues from KISS1R tridimensional model
Panel A: The general case of non-Gly, non-Pro, non-prePro residues. Panel B: The Gly residues, shown with two fold symmetrized contours.
Panel C: The Pro residues with contours.
Panel D: The pre-Pro residues (excluding those that are Gly or Pro) with contours.
seeMOLPROBITY (http://kinemage.biochem.duke.edu)
Figure S1. Rammachandran plots for human KISS1R tridimensional model. Statistics, calculated
with Procheck, showed that 99.0% of the residues in the Ramachandran plot were in the most
favored or allowed regions, and that side-chain stereo parameters were within the range of or
 better than the statistics derived from a set of crystal structures of at least 2.0 Å resolution.
