# Supplementary material for: Two Families with Normosmic Congenital Hypogonadotropic Hypogonadism and Biallelic Mutations in KISS1R (KISS1 Receptor): Clinical Evaluation and Molecular Characterization of a Novel Mutation
Source: PLoS One. 2013 Jan 18;8(1):e53896. doi: 10.1371/journal.pone.0053896 (PMC3548821; doi:10.1371/journal.pone.0053896)
Supplement: Figure S2 — Human KISS1R tridimensional model. Panel A: Tyr313, located in the middle of 7th transmembrane segment, is pointing at the hydrophobic core. The hydroxyl group of the tyrosine residue may form 2 hydrogen bonds with Cys95 and Thr99 both located in the 2nd transmembrane segment and stabilize cohesion of transmembrane bundle. These residues may participate in the docking of kisspeptin. Panel B: Focus on the mutated amino-acid residue: on the left, hydroxyl group of Tyr313 residue probably make hydrogenic bond with Cys95 (2.75 Å) and Thr99 (2.62 Å) (Normal Hydrogenic bond range is 2.7 to 3.2 Å). On the right: Imidazole group of histidine mutant residue in position 313 is not able to form hydrogen bond with Cys 95 and Threonine 99 both located in the 2nd transmembrane segment and therefore impair cohesion of transmembrane bundle. (PPT) [file pone.0053896.s002.ppt]

## Slide 1
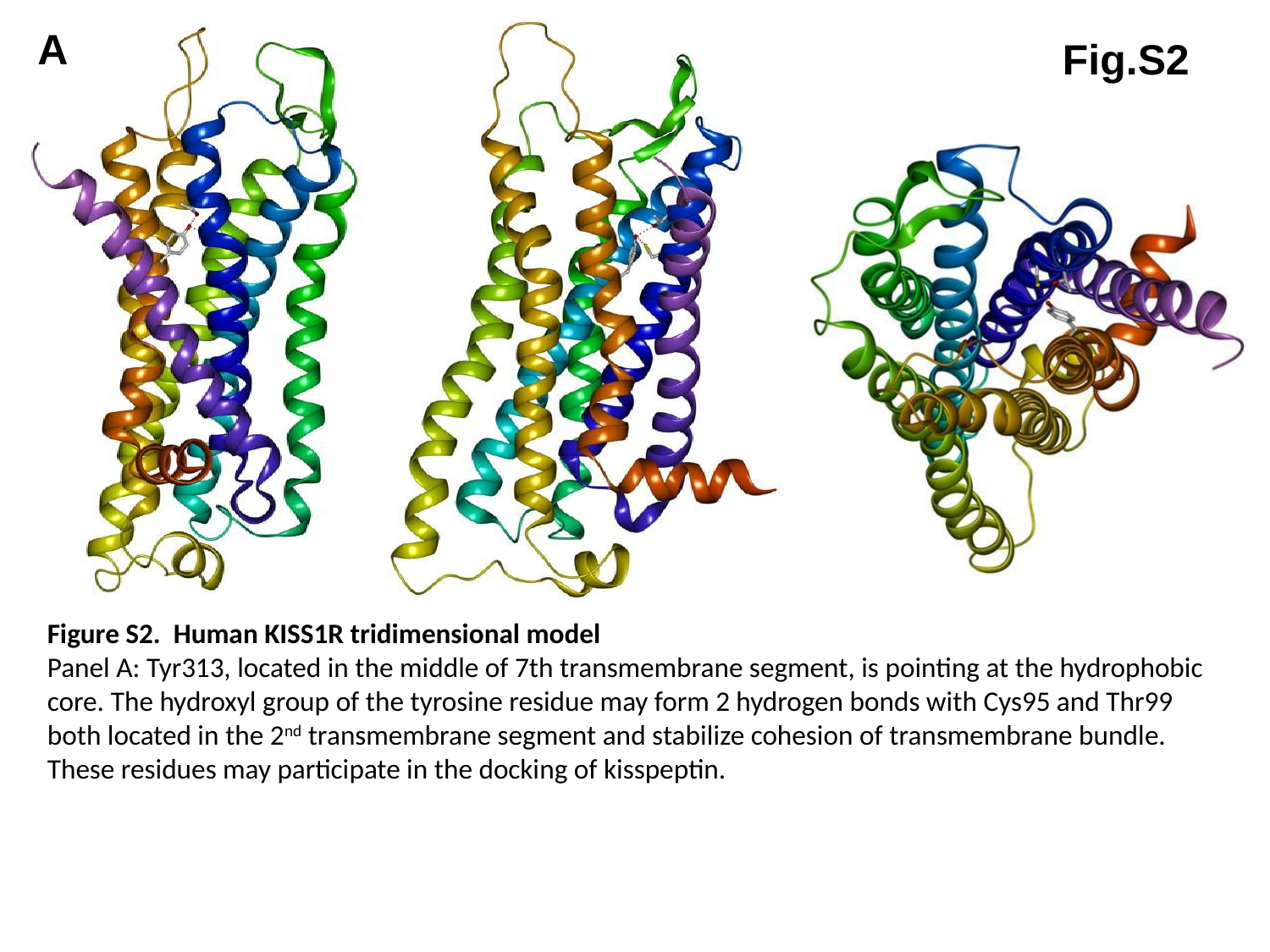

A
Fig.S2
Figure S2. Human KISS1R tridimensional model
Panel A: Tyr313, located in the middle of 7th transmembrane segment, is pointing at the hydrophobic core. The hydroxyl group of the tyrosine residue may form 2 hydrogen bonds with Cys95 and Thr99 both located in the 2nd transmembrane segment and stabilize cohesion of transmembrane bundle. These residues may participate in the docking of kisspeptin.

## Slide 2
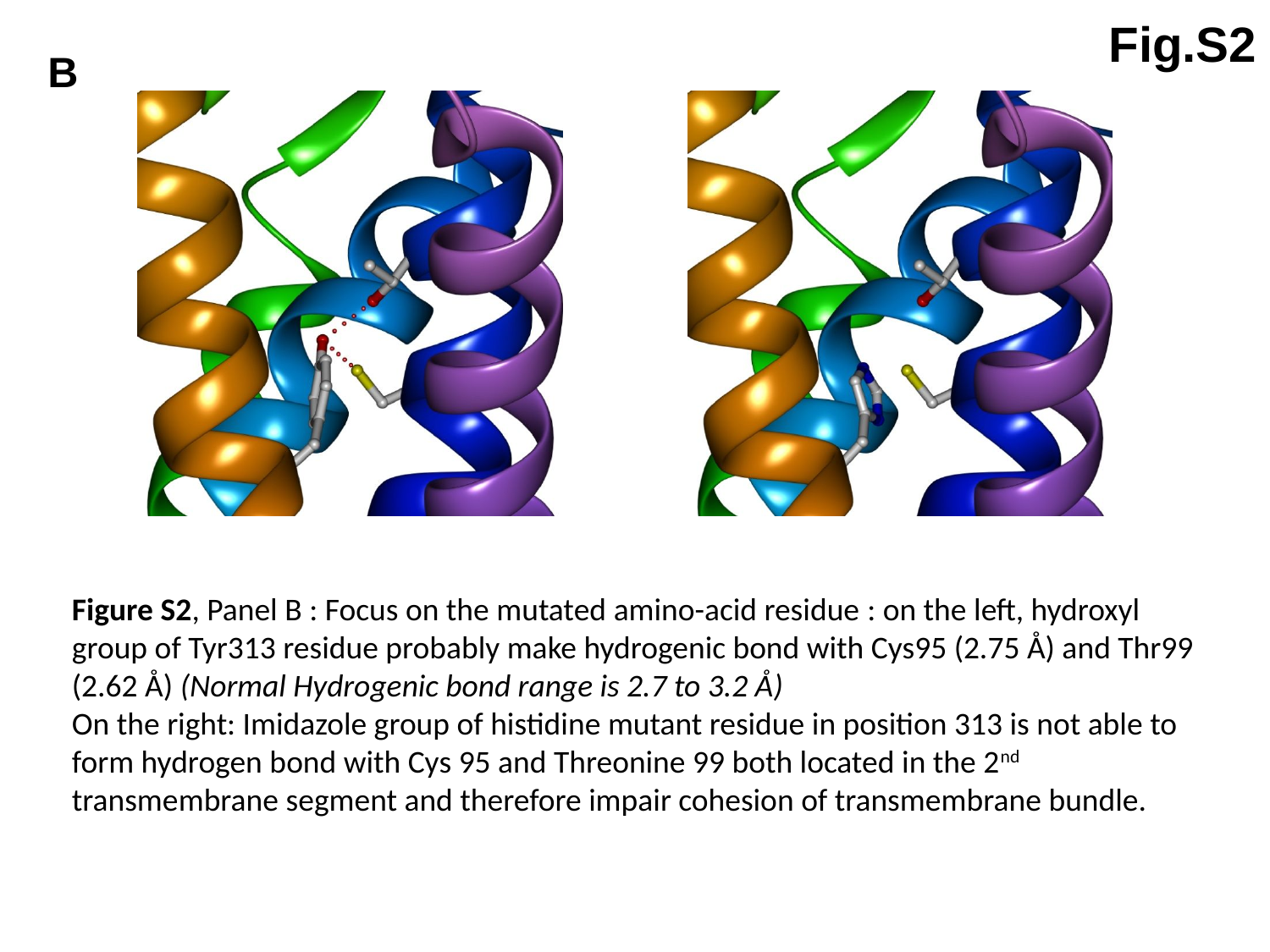

Fig.S2
B
Figure S2, Panel B : Focus on the mutated amino-acid residue : on the left, hydroxyl group of Tyr313 residue probably make hydrogenic bond with Cys95 (2.75 Å) and Thr99 (2.62 Å) (Normal Hydrogenic bond range is 2.7 to 3.2 Å)
On the right: Imidazole group of histidine mutant residue in position 313 is not able to form hydrogen bond with Cys 95 and Threonine 99 both located in the 2nd transmembrane segment and therefore impair cohesion of transmembrane bundle.
